# Potential miRNAs for miRNA-Based Therapeutics in Breast Cancer

**DOI:** 10.3390/ncrna6030029

**Published:** 2020-07-13

**Authors:** Jun Sheng Wong, Yoke Kqueen Cheah

**Affiliations:** 1 Department of Biomedical Science, Faculty of Medicine and Health Sciences, Universiti Putra Malaysia, Selangor 43400, Malaysia; S180053@e.ntu.edu.sg; 2 School of Biological Sciences, Nanyang Technological University, 60 Nanyang Drive, Singapore 637551, Singapore

**Keywords:** breast cancer, microRNAs, cell line, miRNA-based therapeutics

## Abstract

MicroRNAs (miRNAs) are small non-coding RNAs that can post-transcriptionally regulate the genes involved in critical cellular processes. The aberrant expressions of oncogenic or tumor suppressor miRNAs have been associated with cancer progression and malignancies. This resulted in the dysregulation of signaling pathways involved in cell proliferation, apoptosis and survival, metastasis, cancer recurrence and chemoresistance. In this review, we will first (i) provide an overview of the miRNA biogenesis pathways, and in vitro and in vivo models for research, (ii) summarize the most recent findings on the roles of microRNAs (miRNAs) that could potentially be used for miRNA-based therapy in the treatment of breast cancer and (iii) discuss the various therapeutic applications.

## 1. Introduction

According to GLOBOCAN, breast cancer is ranked the second most common cancer in the world. It is the most frequently occurring (24.2%) with the highest cancer fatality among women (15%). Asia (49.6%) has the highest mortality cases for both genders, followed by Europe (22%), Africa (11.8%), Latin America and the Caribbean (8.4%), North America (7.5%) and Oceania (0.77%) [1]. Based on the intrinsic molecular subtypes, breast cancer can be classified into various forms: Luminal A, Luminal B, Basal-like, Her2-enriched, and Normal-like. Particularly, triple negative breast cancer (TNBC) constitutes about 15–20% of all breast cancers [2]. Using transcriptomic profiling, it was identified that 49% of TNBC cases are Basal-like subtypes and 30% are claudin-low subtype [3]. TNBC indicates the deficit expressions of the estrogen receptor (ER), progesterone receptor (PR) and human epidermal growth factor receptor-2 (Her2), which thereby render endocrine or targeted therapies ineffective [2,3]. TNBC is highly heterogeneous and aggressive, with no standard recommended therapy for treatment. On the other hand, these therapies can effectively treat cancer subtypes Luminal A (triple positive), Luminal B (ER+, PR+ and Her2-) and Her2 enriched (ER-, PR-, Her2+). Nonetheless, regardless of all subtypes, hormone resistance and tumor relapse remain the major factors to hamper the effectiveness of therapy for breast cancer.

MicroRNAs (miRNA) are a class of short single stranded, non-coding RNAs (approximately ~22 nucleotides) that play an important role in regulating cancer development and progression [4]. They function by binding to the partial complementary mRNA seed sequence at the 3’ untranslated region (UTR) of mRNA targets [5]. Ultimately, the translation is repressed, or the cytoplasmic mRNA is degraded. Aberrations in miRNA expressions can affect gene expressions that are essential in cellular development, differentiation, proliferation, survival, apoptosis, resistance, and motility. As miRNAs can silence a wide variety of these genes simultaneously, and that the dysregulation of miRNAs are associated with different breast cancer subtypes and tumor malignancy, this makes them an attractive candidate for drug development. This review will summarize the recent findings on the emerging roles of miRNAs and discuss their potential in miRNA-based therapy for breast cancer.

## 2. miRNA Biogenesis and Mode of Action

**Figure 1:**
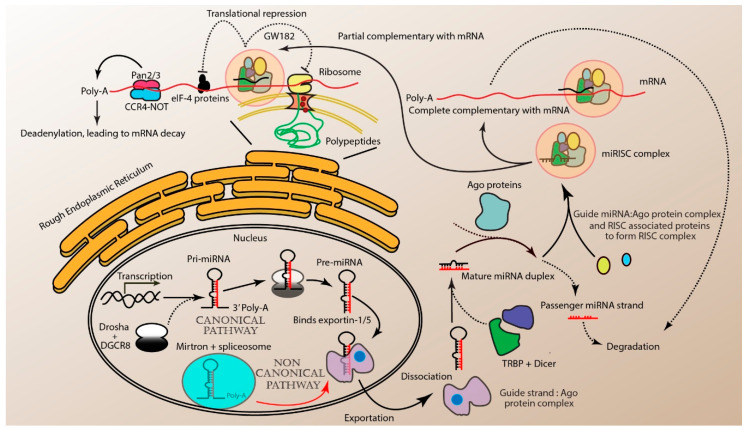
miRNA biogenesis: the canonical pathway begins with the transcription of miRNA genes to form the pri-miRNA. Pri-miRNA is further processed by the Drosha–DiGeorge Syndrome Critical Region 8 (Drosha–DGCR8) complex to produce precursor miRNA (pre-miRNA). Non-canonical pathway, mirtrons bypass the Drosha cleavage and instead, is cleaved by the intron-lariat-debranching enzymes. Both the pre-miRNAs are then transported to the cytoplasm by the Exportin5/RanGTP complex and cleaved to produce mature miRNA. The miRNA duplex consists of guide miRNA (black strand) and passenger miRNA (red strand). The guide miRNA binds the Ago protein and RNA-induced silencing complex (RISC)-associated proteins to form the miRNA-induced silencing complex (miRISC) complex while the passenger miRNA (red strand) is degraded. Guide miRNA facilitates the miRISC complex binding to mRNA targets. Perfect or near perfect complementarity of the guide miRNA to mRNA targets results in the direct cleavage by miRISC. Partial complementarity to the target site can result in translational suppression by interfering with the ElF4A/E/G complex. The miRISC complex binds to 3’-UTR elements of mRNA and recruit PAN2/3 and CCR4-NOT to induce mRNA deadenylation and eventually, its degradation. Alternatively, GW182 proteins can interact with miRISC and induce translational repression.

miRNA synthesis is carried out through canonical and non-canonical pathways (Figure 1). Most miRNAs are processed by the canonical pathway. The first step of the canonical biogenesis pathway is the transcription of genomic DNA by RNA polymerase II to generate primary miRNA (pri-miRNA). Pri-mRNA consists of a stem-loop structure, a methylguanosine cap and may or may not necessarily have a poly-(A) tail [6,7]. It is cleaved by the Drosha–DiGeorge Syndrome Critical Region 8 (Drosha–DGCR8) complex to form a 70–100 nt precursor miRNA (pre-miRNA). Pre-miRNA is then transported to the cytoplasm by the association with Exportin-5/RanGTP complex. Subsequently, RNase III endonuclease Dicer cleaves the terminal loop to form a mature miRNA duplex. This duplex is loaded into the Argonaute family of proteins (AGO1–4). Based on the thermodynamic properties and the degree of complementarity between the miRNA and the AGO protein, one of two miRNA strands will be selected as the guide strand. The guide strand complexes with the Ago protein to form the miRNA-induced silencing complex (miRISC), while the other passenger strand from the duplex is degraded [6,8].

The non-canonical miRNA biogenesis pathway is an alternative for generating miRNAs (Figure 1). It can be classified into Drosha-DGCR8-independent or Dicer-independent systems. These pathways have been thoroughly reviewed by Stavast et al., 2019 and Abdelfattah et al., 2015 [7,9]. Briefly, pri-miRNAs that are encoded in the intron of coding genes, also known as miRtrons, are being processed by nuclear splicing machinery to form shorter hairpin loops that are shorter than the canonical pri-miRNAs. These miRtrons are Drosha–DGCR8-independent and they undergo hydrolysis by debranching enzyme 1 (DBR1) protein. The mirtron-derived pre-miRNAs are then directly translocated to the cytoplasm mediated by Exportin-5 (XPO-5) and bypassed the cleavage by endoribonuclease Dicer. For Dicer-independent systems, short hairpin RNAs (shRNA), small nucleolar RNAs (snoRNAs) and unique miRNAs such as pre-miR-451 are processed and transported similarly to the canonical pathway. They bypass the activity of Dicer and are further processed [5,7,8,9]. Both pathways will eventually lead to the formation of an miRISC complex, consisting of guide miRNA strand and the AGO protein. The guide miRNA is able to bind to the 5′ UTR promotor and coding regions of the corresponding mRNA via complementary base-pairing, known as miRNA response elements (MREs) [5]. Perfect complementarity between the miRNA of the miRISC complex and the MRE of the mRNA target promotes the AGO-dependent mRNA cleavage. Partial complementarity with only 2–8 base pairing, called the seed region, is sufficient for the interaction [10]. The miRISC complex, in this case, promotes the dissociate of eIF4A from the target mRNAs [11]. Furthermore, the miRISC complex together with TNRC6 protein, recruits the PAN2–PAN3 and CCR4–CAF1–NOT deadenylase complexes that remove the 3′ poly-A tail of the mRNA targets and thereby, inhibiting translation [12].

## 3. Cell Lines as In Vitro Model

**Figure 2:**
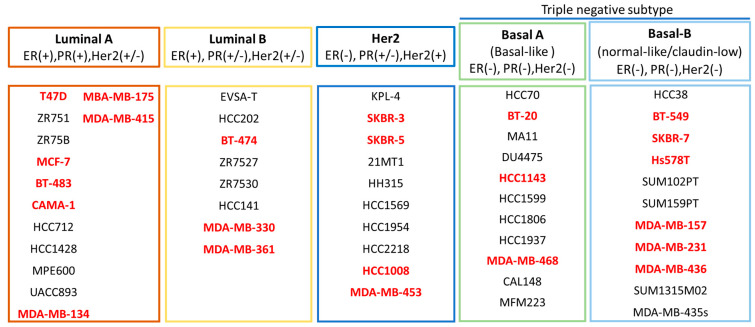
Classifications of the breast cell lines. The cell lines are grouped into Luminal A, Luminal B, Basal-A and Basal B subtypes. Bold red indicates the cell lines that are commonly used for research studies [13,14,15,16].

There are more than 100 breast cancer cell lines that are commercially available. A study by Dai et al., 2017 characterized 84 of these cell lines based on their molecular profiles and genetic compositions [13]. These cell lines were classified into Luminal A, Luminal B, Basal-like, Her2 enriched and Normal-like (Figure 2). Each subtype has distinctive features. Some of the common Luminal A subtype cell lines with ER and PR positive but Her2 negative profile are MCF-7, T47D and MDA-MB-415. The cell lines for the Luminal B subtype with ER and Her2 positive but PR negative are MDA-MB-330 and MDA-MB-361. The HCC1008 cell line is a Her2 subtype which has a status of Her2 positive and a hormone negative profile. Basal-like subtype cell lines are triple negative and can be further classified into A and B, with Basal B being more aggressive than Basal A. For example, MDA-MB-468 and BT-20 are classified as Basal A and SKBR-7, BT-549 and MDA-MB-231 cell lines are Basal B, characterized as a claudin-low subtype [13,14,15]. Notably, each of these subtypes derives from difference sources. For example, MDA-MB-231 and BT-549 are Basal A subtype. The MDA-MB-231 cell line originates from adenocarcinoma while BT-549 from invasive ductal carcinoma [13,14,15]. In addition, the cell lines of the same subtype may also have a different genetic profile, leading to different responses to the same treatment. Thus, it is important to choose the suitable cell lines for an appropriate research study. In addition, the long-term serial passaging of cell lines are also known to alter the key functions of the original cell lines. Therefore, cell-based studies often are accompanied by in vivo studies to determine the functional roles of miRNA in breast cancer [16].

Most of the cellular assays utilize a two-dimensional (2D) monolayer cell culture system by growing cells on coated flat dishes. One of the major drawbacks of this system is the failure to mimic the cellular microenvironment in vivo, such as the lack of cross-talking between the different types of cells within the system, and the absence of extracellular matrix and growth factors [17]. To overcome these limitations, the 3D cell culture was developed. The cells can be grown into 3D spheroids in a suspension medium or embedded within or on the surface of a scaffold or matrix. The spheroid core cells are hidden from the environment and thus, receive less oxygen and nutrients from the medium. This resembles the tumor microenvironment occurring in vivo [18]. To resolve the dissimilarity between the genetic and biological responses of the cell lines and human subjects, animal models were utilized to evaluate the therapeutic efficacy and toxicity prior to clinical trials. Murine is the most extensively employed in vivo model due to the ability to breed rapidly, its susceptibility to multiple human diseases and the fact it is easy to handle. In general, mouse xenograft models can be classified into three main categories: 1) cell-derived xenografts (CDX), 2) patient-derived xenografts (PDX), and 3) the syngenic model (SM). Some of the mice stains that are utilized in CDX and PDX xenograft models are NOD/SCID and nude mice. The CDX transplantation model involves the subcutaneous or orthotropic transplantations of tumor cells such as MDA-MB-231 and MCF-7 into immunocompromised mice to investigate the metastatic potential and cancer progression [19,20]. This model is commonly used for gene function validation and to evaluate the effectness of anti-cancer drugs. For a PDX transplantation model, primary human breast carcinoma derived from patients are characterized by institutions or organisations and can be transplanted into immunodeficient mice [21]. Compared to the CDX model which uses cancer cell lines, the PDX model maintains the characteristic and genomic signature of the human tumors and therefore, providing a more accurate reflection of drug tolerance and the sensitivity of a patient during drug screening. The SM model transplants murine cancer cells such as Py2T and 4T1 into immune-competent mice (e.g., FVB/N and BALB/c). This animal model is primarily used for studying the interaction between tumors and immune cells, and the effects of treatment involved in the immune system such as immunotherapies. In addition, transgenic mice can be modified by knocking out certain genes to unravel the underlying molecular mechanisms. Toru et al., 2019 also developed a novel C57BL/6 mouse model of the bone metastases of breast cancer by using Py8119 and PyMT-BO1 (bone metastatic) cells. These mice allow the researchers to study the microenvironment and the immune system of the bone metastases of breast cancer [22]. Taken together, these well established in vitro and in vivo models are important for the validation and evaluation of the safety and the efficacy of drugs, and understanding the underlying mechanisms regulating cancer progression.

## 4. Roles of miRNAs in Cancer

**Figure 3:**
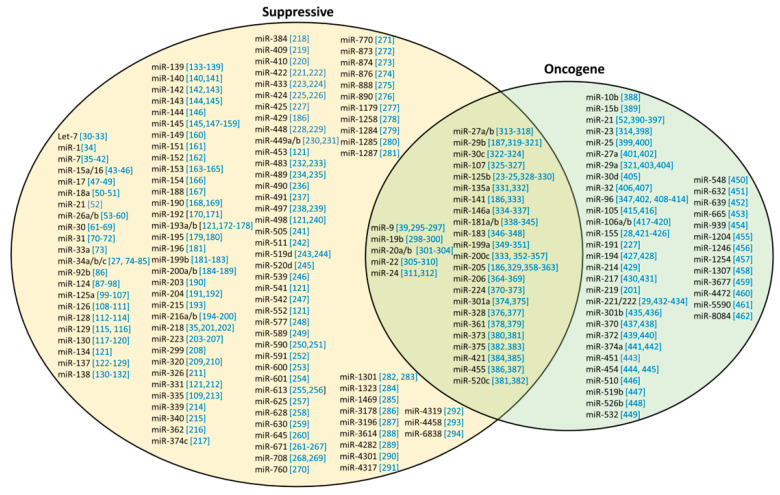
Roles of miRNAs. Summary of all the articles published from 2018 to 2020 in PubMed Database [30,31,32,33,34,35,36,37,38,39,40,41,42,43,44,45,46,47,48,49,50,51,52,53,54,55,56,57,58,59,60,61,62,63,64,65,66,67,68,69,70,71,72,73,74,75,76,77,78,79,80,81,82,83,84,85,86,87,88,89,90,91,92,93,94,95,96,97,98,99,100,101,102,103,104,105,106,107,108,109,110,111,112,113,114,115,116,117,118,119,120,121,122,123,124,125,126,127,128,129,130,131,132,133,134,135,136,137,138,139,140,141,142,143,144,145,146,147,148,149,150,151,152,153,154,155,156,157,158,159,160,161,162,163,164,165,166,167,168,169,170,171,172,173,174,175,176,177,178,179,180,181,182,183,184,185,186,187,188,189,190,191,192,193,194,195,196,197,198,199,200,201,202,203,204,205,206,207,208,209,210,211,212,213,214,215,216,217,218,219,220,221,222,223,224,225,226,227,228,229,230,231,232,233,234,235,236,237,238,239,240,241,242,243,244,245,246,247,248,249,250,251,252,253,254,255,256,257,258,259,260,261,262,263,264,265,266,267,268,269,270,271,272,273,274,275,276,277,278,279,280,281,282,283,284,285,286,287,288,289,290,291,292,293,294,295,296,297,298,299,300,301,302,303,304,305,306,307,308,309,310,311,312,313,314,315,316,317,318,319,320,321,322,323,324,325,326,327,328,329,330,331,332,333,334,335,336,337,338,339,340,341,342,343,344,345,346,347,348,349,350,351,352,353,354,355,356,357,358,359,360,361,362,363,364,365,366,367,368,369,370,371,372,373,374,375,376,377,378,379,380,381,382,383,384,385,386,387,388,389,390,391,392,393,394,395,396,397,398,399,400,401,402,403,404,405,406,407,408,409,410,411,412,413,414,415,416,417,418,419,420,421,422,423,424,425,426,427,428,429,430,431,432,433,434,435,436,437,438,439,440,441,442,443,444,445,446,447,448,449,450,451,452,453,454,455,456,457,458,459,460,461,462], using the search engine with the keywords “breast cancer” and “miRNA”. The articles that are relevant for the discussion in this review are included. The functions of miRNAs are divided into 3 categories: tumor suppressive (beige circle), oncogenic (green circle), or both (beige/green circle). The majority of the miRNAs fall under tumor suppressive.

The dysregulation in miRNA expressions is associated with the development of cancer and resistance to cancer therapy. Although miRNA is a promising alternate therapeutic for breast cancer, literature on the roles of miRNAs as tumor suppressor miRNAs (ts-miRNA) and oncogenic miRNAs (onco-miR) have been inconsistent. Recent studies have also shown that certain miRNAs that have previously been known to be solely oncogenic or tumor suppressive, are now having a dual function in breast cancer. For example, miR-125b has been a well recognized ts-miRNA that can target ENPEP [23], CK2-α [23], CCNJ [23], MEGF9 [23], MMP11 [24] and KIAA1522 [25] genes and inhibit cancer progression in MDA-MB-231 cells. However, a recent study by Nie et al., 2019 demonstrated that miR-125b can also act as an oncogenic onco-miRNA through the Adenomatous polyposis coli (APC)-mediated Wnt/β-catenin pathway [26]. In addition, miRNAs can influence the expressions of multiple genes and therefore, diversify the effects on cancer. Hence, the selection of miRNAs for therapeutic approaches should be handled with caution. In this section, the emerging roles of existing miRNAs and potential miRNAs for miRNA therapeutics will be discussed. The well established miRNAs such as miR-34 [27], miR-155 [28], miR-221/222 [29] will be excluded from discussion. The potential miRNAs selected for discussion are based on the following criteria (Figure 3):

1. Sole function as onco-miR or ts-miR;
2. miRNA signatures (miRNA profiles in tumor stages and subtypes);
3. Validation of miRNA functions (loss/gain of function);
4. Multiple reports supporting its functions (same cell types/subtypes);
5. Pharmacological studies—non-toxic (in vitro and in vivo studies);
6. Sensitizing cancer cells to standard therapy (optional).

### 4.1. Tumor-Suppressive miRNAs (ts-miRs)

**Figure 4:**
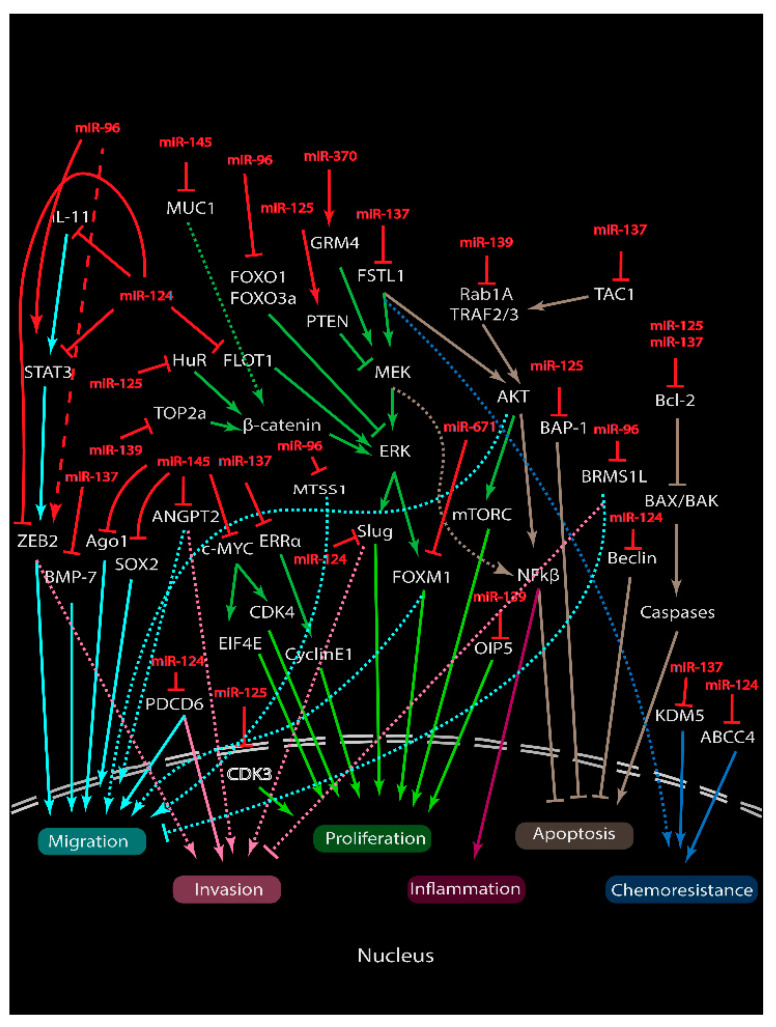
Roles of miRNA in cell signaling regulating tumorigenesis. Oncogenic miR-370 upregulates GRM4 expression and miR-96 regulates the expression of FOXO, MTSS1, BRMS1L and ZEB1 proteins. Both resulted in the promotion of proliferation and invasion. Tumor suppressive miR-124, miR-125, miR-137, miR-139, miR-145 and miR-671 can suppress oncogenic targets and inhibit cancer progression. miR-124 can bind Slug, STAT3, ZEB2, PDCD6, Beclin-1, FLOT1, IL-11 and ABCC4. miR-125 can interact with HuR, BAP-1, PTEN, Bcl-2 and CDK3. miR-137 targets FSTL1, BMP-7, ERRα, Tac1 and KDM5. miR-139 can binds TOP2a, RAB1A and OIP5. miR-145 directly inhibits MUC1, ANGPT2, c-Myc, Sox-2 and Ago1. miR-671 regulates FOXM1 expression. All, in turn, result in the regulation of cancer progression by suppressing the cancer hallmarks including chemoresistance.

ts-miRs are underexpressed in breast cancers and can directly or indirectly suppress the expression of oncogenic genes, preventing cancer development and malignancy (Figure 3 and Figure 4).

**miR-124** expression negatively correlates to pathological breast cancer tissues. miR-124 level in malignant tissues (Grade 3) was significantly lower than that of the less severe (Grade 2) [87]. Another three studies also showed a further reduction of the miR-124 expression in distant metastases of the lymph node and bones compared to the non-metastatic ones [88,89,90]. Breast cancer patients who have a higher miR-124 expression have a better overall survival rate than the patients with low miR-124 expression [91]. In addition, the investigation of miR-124 expression in nine breast cancer cell lines showed that the miR-124 level was the lowest in the highly metastatic MDA-MB-231 (TNBC) amongst the others [87]. Slug is a master regulatory transcription factor for the epithelial–mesenchymal transition (EMT) and is responsible for downregulating E-cadherin and upregulating vimentin. The miR-124 direct repression of Slug resulted in the reduction of the metastatic potential of MDA-MB-231 cells. Shi et al., 2019 and Ji et al., 2019 also showed that miR-124 could negatively regulate the ZEB2 [92] and STAT3 [93] expression via 3’ UTR interaction and inhibit the viability and invasion of breast cancer on TNBC cell lines. PDCD6 is another direct target that is found to regulate EMT and cell motility. Reconstituting PDCD6 expression impaired the tumor suppressive function of miR-124 [91]. Li et al., 2013 revealed that flotillin-1 (FLOT1) is also suppressed by miR-124 and the inverse correction between miR-124 and FLOT1 levels was associated with tumor stage and progression [88]. Zhang et al., 2016 and Wang et al., 2016 also confirmed that Beclin-1 (autography-related protein and Cbl (uiquitin protein ligase) were controlled by miR-124 and the negatively regulated progression of breast cancer [94,95]. Metastasis can also be due to the change in modulating factors specific to distant sites. Cai et al., 2018 demonstrated that miR-124 can bind to IL-11 and negatively modulate its expression in vivo and in vitro. The reduction of active IL-11 by miR-124 regulates osteoclastogenesis and suppresses metastasis to the bone [89]. These findings proved that miR-124 is important in regulation the invasion and metastatic potential of malignant breast cancer. Apart from this, miR-124 was also investigated on their role in chemoresistant breast cancer. As aforementioned, miR-124 could interact with STAT3 and another study by Liu et al., 2019 demonstrated that miR-124 could sensitize doxorubicin-resistant (DOX-R) cells by the regulation of the STAT3 and HIF-1 signaling pathway [96]. STAT3 expression was upregulated in DOX-R breast cancer stem cells as shown by Western blot. The overexpression of miR-124 resulted in the suppressed invasion and proliferation of DOX-R MCF-7 while co-transfection with STAT3 reversed the effect. miR-124 mediated and downregulated STAT3-induced HIF-1 expression in MCF-7 using Western blot and sensitized DOX-R MCF-7 to doxorubicin. Another study by Hu et al., 2019 also showed that miR-124 targets ABCC4 and sensitizes chemoresistance MCF-7 to DOX [97]. Large tumor size and metastatic tumor also correlate with the increased ABCC4 protein level and are reduced in miR-124 level. By overexpressing miR-124 or inhibiting active ABCC4, this resulted in a decreased in cell proliferation, invasion and migration of DOX-resistant MCF-7. In addition, the combined effects of ABCC4 and miR-124 synergistically inhibit these effects. These studies demonstrate that miR-124 could be used as a single agent or potentially, in combination with other therapies to synergize the anti-cancer effects on chemoresistant breast cancer.

**miR-125a** belongs to one of the three homologs of the miR-125 family with another two—hsa-miR-125b-1 and hsa-miR-125-2. It acts as a tumor suppressor and its expression is significantly downregulated in breast cancer tissues and cell lines. The lower expression of miR-125a is associated with lower overall free survival (OFS) and progression-free survival (PRS) [99]. Patients with metastasized miR-125-positive lymph nodes have a worse survival rate compared to miR-125 negative lymph nodes. HuR is an RNA binding protein (RBP) that regulates the transcription of oncogenes. miR-125’s direct repression of HuR through the 3′ UTR resulted in the inhibition of cell growth in breast cancer cell lines [100]. Fyn is a tyrosine protein that can induce Ras/PI3K/Akt signaling and promote cancer pathogenesis and drug resistance. miR-125a binds Fyn and regulates its expression and activity. The inhibition of Fyn induced cell cycle arrest and reduced migration in breast cancer [101,102]. BAP-1 is also another target of miR-125 and BAP-1 suppression resulted in the cell apoptosis of MCF-7 and MDA-MB-468 cells [103]. miR-125 also helps to regulate gene expression involving cancer progression. The overexpression of miR-125a-5p increases PTEN expression and significantly reduces phosphorylated MEK and ERK expressions and suppression in cell proliferation and migration in MCF-7. Using Hoechst staining, flow cytometry and Western blot, miR-125a-induced apoptosis was shown to be mediated by caspase-3 cleavage and decreased in the Bcl-2 level [104]. Two recent papers also demonstrated the role of miR-125 as a drug-resistant sensitizer. Zheng et al. found that miR-125a can directly interacts with CDK3 and facilitates CDK3-dependent inhibition of the transcriptional activity of ERα by decreasing the ERα Ser118 phosphorylation. This resulted in suppressed cell proliferation and colony formation in ER-positive breast cancer [105]. miR-125a is downregulated in tamoxifen (TAM)-resistant MCF-7 and miR-125 overexpression re-sensitized the cells to TAM in vitro and in vivo. The other study by Ninio-Many et al., 2020 revealed that combination treatment with miR-125a-3p and Trastuzumab (TRA) could synergistically suppress cancer progression [106]. miR-125a induced an increase in cell surface ErbB2 and re-sensitized MDA-MB-231 (Her2 (-)) to TRA, which targets the ERBB2 (Her2) receptor. Co-treatment with miR-125 and TRA synergistically suppressed cell migration and tumor growth in vitro and in vivo. These studies indicate that miR-125a not only targets Her2 (+) breast cancer and could also overcome resistant cancer when used in combination with chemotherapeutic drugs.

**miR-137** is significantly downregulated, specifically in the TNBC subtype of breast cancer. miR-137 binds with the 3′-UTR of BCL-11a and downregulates its expression and inhibits the BCL11a-DNMT-1 mediated proliferation in MDA-MB-231 cells but not in MCF-7 [122]. miR-137 also represses the expression of ERRα through the downstream effect of cyclinE1 and WNT11 proteins which are involved in proliferation and migration [123]. Another four studies also showed that DUSP4 [124], Del-1 [125], Tac1 [126], KDM5 [127] histone demethylases were direct targets of miR-137 and were overexpressed in TNBC cells. The suppression of these targets by miR-137 promote cancer progression. In addition, the increase in miR-137 level alleviates resistant to cisplatin (Cis) and DOX significantly by inhibiting the expression of DUSP4 [124] and follistatin-like 1 (FSTL1) [128] via integrin β3/Wnt signaling in TNBC. On the contrary, mir-137’s direct inhibition of BMP7 could enhance the epithelial–mesenchymal transition (EMT) potentials in MCF-7 [129]. The cell lines mainly used in these studies are the MCF-7 of Luminal A subtype and MDA-MB-231 of the Basal subtype. These studies suggest that miR-137 consistency and specifically inhibit TNBC progression and susceptibility to chemotherapeutic drugs.

**miR-139** is significantly underexpressed in the tumor. Amongst the subtypes, TNBC patients show a higher downregulation of miR-139 compared to ER/PR or Her2-expressing ones [133]. miR-139 is associated with tumor aggression. A recent report indicated that miR-139 was dramatically downregulated in an aggressive grade 3 tumor with a hormonal negative (especially, TBNC) status, with poor clinical outcome [134]. An interesting target by miR-139 is the TOP2a gene that encodes for the Topoisomerase 2a protein, which is important in transcription and cell replication. A high TOP2a level was observed in Luminal A and B subtypes but not in Her2-positive or Basal-like patients. The direct suppression of TOP2A by miR-139 in MCF-7 and T47D suppresses cell proliferation [135]. These studies indicated their functional role in development and metastasis. The miR-139 suppression of RAB1A, which belongs to the Ras oncogene family, is associated with cell growth, migration, and invasion [136]. Another miR-139 target is the OIP5 oncogene. The inhibition of OIP5 resulted in the retardation of cell proliferation through miR-139-5p/Notch1 [137]. Recently, a study by Pajic et al., 2018 showed that miR-139-5p could sensitize breast cancer to radiotherapy (RT). MicroRNA microarray profiling showed that miR-139 was overexpressed in the non-relapse breast cancer patients compared to patients with relapsed breast cancer (surgery + RT) [138]. Introducing the miR-139 mimic into MCF-7, followed by radiation exposure significantly enhanced cell death. A luciferase reporter assay demonstrated that miR-139 could negatively regulate MAT2A, POLQ, TOP1, and TOP2A genes which are critical in DNA repair and antioxidant. Moreover, combination treatment using RT with miR-139-5p mimics significantly suppressed proliferation (low Ki-67 cells) and DNA repair (high Phospho-H2AX) and sensitizing the cells to radiotherapy in vivo. Moreover, miR-139-5p can also mediate Notch1-induced chemosensitivity to docetaxel in vitro [139]. These studies indicate that miR-139 as a potential therapeutic agent could be used in single or combination regime with other therapies in treating breast cancer.

**miR-145** is significantly downregulated in the metastatic breast cancer specimens and cell lines compared with normal breast tissues. The expression of miR-145 was lower in patients with metastatic cancer as compared to non-metastatic cancers. miR-145 is epigenetically downregulated by hypermethylation in metastatic breast cancer [147,148]. In the same study, the researchers also showed that miR-145 can directly target angiogenic factor ANGPT2 and suppress tumor metastasis. TP-53 activation can stimulate miR-145 expression via the interaction between the p53 response element and the promoter region of miR-145 [149]. The upregulation of miR-145 directly silences oncogene c-Myc and c-Myc downstream target genes, eIF4E, and CDK4, resulting in the suppression of tumor growth. Increasing evidences have shown that miR-145 suppresses the metastatic potential of early stage cancer. The ectopic expression of miR-145 on metastatic cell lines has no significant effect on cell proliferation. The silencing of mucin 1 (MUC1) gene, which in turn downregulates β-catenin, cyclin D1 and cadherin 11, causes the invasiveness and metastasis of these cells to be suppressed [150]. In contrast, other studies showed that miR-145 can directly bind SOX-2 [151], TGFβR2 [152] and TGF-β1 [153] and inhibit cancer progression in metastatic MDA-MB-231 and MCF-7 cells. García-García et al., 2019 reported that miR-145-5p restoration induced cell death and sensitized BT-20, MDA-MB-231, MCF7 and SKBR3 cells to Cis treatment [152]. In addition, after treatment with the chemotherapy regimen, patients with low level of miR-145 have a higher disease-free survival rate as compared to patients with a high level of miR-145. Götte et al., 2010 also demonstrated that miR-145 modulates the actin cytoskeleton remodeling and homogenizes cortical actin distribution, nuclear rotation, and migration via the direct interaction with 3′UTR of JAM-A and fascin in MDA-MB-231 cells [154]. In a similar context, the expression of SMAD3 [148], DR5 [148], BRCA2 [148], HBXIP [155] and RTKN [156] directly silenced by miR-145 inhibits tumor metastasis. Angiogenesis is one of the hallmarks of epithelial–mesenchymal transition (EMT). Zou et al., 2012 showed that miR-145 can also negatively regulate N-RAS and VEGF-A post translationally and inhibit tumor growth and angiogenesis in vivo [157]. Meanwhile, the miR-145-induced expression of the Ago2 protein inhibits the migration of MDA-MB-231 in an Ago2-dependent manner [158]. The gene expression profiling of 1538 transcripts also revealed that 698 of the transcripts are downregulated and 840 are upregulated in the presence of miR-145-5p and Ago1 expression. Of which, the genes regulating cell proliferation, chemoresistance and migration were downregulated, suggesting its crucial role in regulating anti-cancer gene expression. miR-145 is one of the most promising candidates for developing miRNA-based therapy targeting chemoresistance TNBC.

**miR-671-5p** is among the most recently researched miRNAs that was observed to be downregulated in breast cancer patients with invasive ductal carcinoma and ductal hyperplasia [261]. microRNA profiling of interstitial fluid of breast tumors showed that the expression of miR-671 was downregulated in Luminal A, Luminal B and TNBC subtypes, but upregulated in the Her2 subtype [262]. It has the sole function as tumor suppressor by targeting FOXM1 and suppresses the VEGF/TGFB-mediated of proliferation and invasion, and BRIP1/Rad51-mediated repair mechanism [263,264]. Tan et al., 2019 also demonstrated that miR-671-5p sensitizes breast cancer cells to UV, Cis, paclitaxel (PAX) and epirubicin (Epi), but not 5-Fu [263]. In addition, the miRNA profiling of chemoresistant subline showed that the expression of miR-671-5p was up by 2-fold in docetaxel-resistant cells but not in epiadriamycin and vinorelbine-resistant cells [266,267].

### 4.2. Oncogenic miRNAs (onco-miRs)

Onco-miRs are overexpressed in breast cancers and can directly or indirectly suppress the expression of tumor-suppressive genes, leading to malignancy (Figure 3 and Figure 4).

**miR-96** expression is elevated in non-malignant and malignant breast cancer cell lines and patients [347,402,407,408,410,411,412,413,414]. Particularly, the miR-96 level was dramatically higher in breast cancers with Her2-enriched subtype, followed by Basal, Luminal A and Luminal B subtypes [347]. This miRNA has shown to be involved in promoting cell proliferation, tumor invasion and epithelial–mesenchymal transition (EMT). FOXO1 and FOXO3a are a tumor suppressive transcriptional factor that can bind to mitogen such as estrogen receptor α and cyclin-dependent kinase inhibitors (CDKi) and regulate cell growth and survival. Guttilla et al., 2009 and Lin et al., 2010 showed that the miR-96 direct suppression of FOXO1 [402] and FOXO3a [408] suppressed the expression of downstream CDKi targets, p27(Kip1) and p21(Cip1), which in turn, upregulates cyclin D1. Notably, the study by Hong et al., 2016 also showed that miR-96 overexpression leads to an increase in the CDK6 and CDK4 mRNA levels [409]. These resulted in the decrease in the cell proliferation of breast cancer cell lines including MCF-7, T47D and MDA-MB-231 [402,408,409]. Following up with these studies, Shi et al., 2017 further demonstrated that miR-96-induced the inhibition of FOXO1 and was also able to suppress autophagy and apoptosis, and promote the proliferation, migration, and invasion in MCF-7 and MDA-MB-231 cells [410]. Since then, there were five papers published to identify its targets and to support its role in EMT. These researchers showed that miR-96 was able to interact with protein tyrosine phosphatase PTPN9 [409], metastasis suppressor-1 (MTSS1) [412], breast cancer metastasis suppressor 1-like (BRMS1L) [413] and growth hormone receptor (GHR) [413] and ZEB1 [414]. The overexpression of miR-96 downregulates these targets and enhances metastatic potentials in breast cancer cells. In addition, the xenograft of MCF-7 harboring pri-miR-96 into the mammary fat pad of mice displayed local infiltration into the muscle and distant spreading into the blood and lymph vessels [413]. One study by Moazzeni performed target screening and showed that ATP-binding cassette transporter A1 (ABCA1) was a target of miR-96. ABCA1 protein is involved in suppressing apoptosis and drug resistance [39]. This indicates that miR-96 may play a regulatory role in the chemosensitization of the breast cancer.

**miR-370** is an oncogenic miRNA that is upregulated in patients with breast cancer. High miR-370 expression is associated with metastasis of lymph nodes, perineural invasion and the advanced stage of the breast cancer [437,438]. Recent studies have slowly revealed that miR-370 has a sole function as a ts-miRNA. Huang et al., 2019 showed that with the overexpression of miR-370, the proliferation and colony formation ability of low miR-370 expressing MCF-10A cells were being enhanced [436]. The knockdown of miR-370 reduced these effects in MDA-MB-231 in vitro and in vivo. WNK3, a key regulator of cellular physiology, was also identified as a target of miR-370 using a luciferase report assay. The overexpression of WNK2 suppressed cell proliferation and colony formation in miR-370 transfected MCF-10A cells [437]. Another study by Xiao et al., 2019 demonstrated that the miR-370 binds and negatively regulates the glutamate receptor GRM4 expression using the dual luciferase report assay [377]. The activity of Firefly/Renilla driven by the GRM4 promoter was reduced while the mutated miR-370 vector rescued the inhibitory effect of the luciferase activity. When miR-370 was transfected into stable MDA-MB-231 expressing GRM4, the invasive capability was enhanced with the increased number of colonies formed. Notably, although both studies showed that miR-370 was upregulated in MDA-MB-231 cells in comparison to the MCF-10A cells, the expression of miR-370 in MCF-7 and SK-BR3 was inconsistent. Apart from looking into the functionality of miR-370 in tumor progression, a previous study by Lv et al., 2014 showed that miR-370 expression was downregulated in MCF-7/DOX cells and chemoresistance specimens from patients with breast cancer [438]. Unfortunately, no studies to date have focused on the role of miR-370 in regulating resistance to neoadjuvant chemotherapy.

Apart from the above eight miRNAs, there are another five emerging miRNAs with therapeutic potential, namely miR-26a, miR-193a/b, miR216a/b, miR223 and miR-1246. Their interacting partners and effects on breast cancer are summarized in Table 1.

Briefly, these four ts-miRNAs could interact with multiple protein partners and suppress the oncogenic properties of breast cancers; 1) miR-26a binds to RNF6 [53], CHD1 [54], GREB1 [54], KPNA2 [54], metadherin [55] and MCL-1 [56], 2) miR-193a/b to DDAH1 [172], PTP1B [173], MORC4 [174], WT1 [175], RAB22A [176], uPA [178], 3) miR-216a/b to TLR4 [194], PKC-α [195], PAK2 [196], SDCBP [197], P2X7R [198] and HDAC8 [199] and 4) miR223 to caprin-1 [203] and STAT5A [206]. Particularly, miR-26a, miR-193a/b and miR-223 could re-sensitize breast cancer cell lines to chemotherapeutic drugs. Tormo et al., 2017 showed that the co-treatment with Tra and siRNA targeting miR-26a suppressed CCNE2 expression and re-sensitized the induced resistant BT-474 cells (Luminal B subtype) [57]. miR-26a/b could also negatively regulate ERBB2 expression post transcriptionally. The forced expression of miR-26a/b decreased the cell viability of TAM-resistant MCF-7 [58]. To investigate the role of miR-193b on drug resistance, Long et al., 2015 first established a DOX-resistant (DOXR) MCF-7 cell line and they showed that the miR-193b expression was significantly downregulated. Using Annexin V/PI staining, the apoptotic effect induced by DOX was greatly enhanced after transfecting miR-193b mimic into DOXR MCF-7 [177]. One of the interesting features of miR-223 is the re-sensitizing effect of broad-spectrum chemotherapeutic drugs on breast cancer cell lines. Two studies by Pinatel et al., 2014 and Sun et al., 2016 showed that the overexpression of miR-223 enhanced the HAX-1-induced anti-cancer effect of DOX and Cis [205], as well as the STAT-5A-induced susceptibility to PTX in MDA-MB-231 cells [206].

On the other hand, the onco-miR-1246 is significantly upregulated in metastatic and drug-resistant breast cancer [266,456]. A recent study by Li et al. indicated that miR-1246 enhances CyclinG2-mediated proliferation, migration and chemoresistance to DOX, Epi and gemcitabine by targeting the expression of Cyclin G2 in breast cancer [456]. However, more studies would have to be conducted to confirm the roles of miR-1246 in drug resistance and cancer progression.

These findings indicate that the inhibition of miR-1246 and the reconstitution of miR-26a, miR-193a/b, miR-216a/b and miR-223 are promising strategies for chemoresistant and metastatic breast cancer.

## 5. miRNA-Based Therapeutics

Currently, there are two strategies to miRNA therapeutics [463,464]; 1) directly, by inhibiting onco-miRs using miRNA antagonists, or indirectly, by utilizing miRNAs or non-miRNA targets that are known to downregulate the specific onco-miRs, and 2) to restore the loss-of-function of ts-miRNAs using ts-miR mimetics. Table 2 shows a summary of the different types of miRNA-based therapies for breast cancer treatment.

### 5.1. miRNA Suppression (Synthetic miRNA-Induced Inhibition)

Because miRNA is a single stranded mRNA and these are exposed to a harsh environment within the cells, the use of synthetic oligonucleotides has been modified to enhance stability, target affinity, and promote cellular uptake. miRNA inhibition focuses on suppressing the overly expressed onco-miR in breast cancer treatment. Synthetic oligonucleotides that are commonly used include locked nucleic acid (LNA), antisense anti-miR oligonucleotides (AMOs) and miRNA sponges [464]. These modifications are often used in inhibition studies to elucidate the roles of miRNAs in cancer.

The logic behind AMOs is to use a sequence that is antisense to their target miRNA, which could result in an efficient and irreversible silencing of the targeted miRNA. They are chemically modified at the C2 carbon of the sugar molecule with a methylated hydroxyl group (2′-OMe RNAs). A new generation of AMOs adds N, N-diethyl-4-(4-nitronaphthalen-1-ylazo)-phenylamine (ZEN) at the 5′- and 3′ ends of the 2′-OMe oligonucleotide to enhance its efficiency and protect itself from nuclease and reduced toxicity. Other modifications include the following five:

1. Addition of methoxyethyl group at the RNA 2′-OH (2-MOE);
2. Addition of fluorine 2′-hydroxyl group at C2 carbon of the sugar group (2′-F);
3. Substitution of oxygen of the phosphate backbone to sulfur to form phosphonothioate linkage;
4. Substitution of phosphate with the uncharged phosphonodiamidite group to form phosphorothioate linkage, known as phosphorodiamidate morpholino oligomers (PMOs);
5. Substitution of phosphate backbone with a pseudo-peptide polymer (N-(2-aminoethyl) glycine) to form an uncharged synthetic DNA, known as peptide nucleic acid (PNA).

Commercial companies utilize the combination of several modifications to generate ts-miR inhibitory oligos. For example, the antagomir (inhibitor) from GenePharma was modified with cholesterol at the 3′ end, and the addition of 2-OMe modified bases and four thiol modifications at the 3′ end [473]. Wang et al., 2017 showed that transfecting with the modified ts-miR-451 antagomir from the GenePharma company rescued the miR-451 suppressive effect in cancer progression and metastasis in vivo and in vitro [443]. Apart from this modification system, miRNA sponges are exogenous competitive inhibitors with multiple tandem binding sites that have strong affinity to the miRNA of interest. This would abolish the miRNA/mRNA interaction. Chemically modified AMOs are generally expensive and have a more off-target effect, albeit being effective as silencers in in vitro studies. Several studies have combined several modification systems together to enhance the anti-cancer effect by the mean of increasing the structure stability and prolong the half-life of the miRNA, with the aim to reduce off-target effects within the cells. One study by Gao et al., 2015 compared the anti-cancer effect of PEI-PLL/miR21-Sponge and PEI-PLL/miR-21-AMO in MCF-7 cells [465]. Both methods induced a significant reduction in cell viability via upregulating the PDCD4 expression, which in turn activated a caspase-3-dependent apoptosis pathway. Notably, PEI-PLL/miR21-Sponge displayed a higher anti-cancer effect when compared to the AMO group. This enhanced effect was due to the prolonged transfection effect by PEI-PLL and that sponge-miR21 plasmid may have a more stable structure than the AMO oligonucleotide. One of the major downsides of miRNA antagonists is the incomplete and temporal knockdown of target miRNAs. Recently, the CRISPR/Cas9 system was developed to effectively overcome these limitations by permanently inducing the gene knockdown of miRNAs in cell lines. This system comprises of a Cas9 nuclease that cleaves a specific DNA site next to a protospacer adjacent motif (PAM) and a guide RNA (gRNA) that facilitates the Cas9 to the specific region, leading to gene-knockout. In a recent study, Hannafon et al. showed that CRISPR/Cas9-induced knockout specifically repressed the targeted miR-23b/27b expression, with minimal disruption to adjacent miRNA precursors in MCF-7 cells [314]. This genetic depletion of the oncogenic miRNAs effectively suppressed tumor growth in vitro and in vivo.

### 5.2. miRNA Replenishment (Delivery Systems)

miRNAs are single-stranded small RNAs that are highly unstable, thus naked RNAs are prone to nuclease degradation before reaching their destination. Safe and specific miRNA carriers have been developed to overcome these problems [463]. The criteria for an ideal delivery system include the protection of miRNAs from degradation; the facilitation of cellular uptake; and being bioinert, biocompatible and non-immunogenic. miRNA delivery systems can be classified into viral and non-viral systems (Table 2). A viral system uses viral vectors such as lentivirus, adenovirus, retrovirus, and adeno-associated virus (AAV). Each of these viral vectors possesses unique characteristics and properties that are meant for different purposes as a delivery vehicle. The miRNA cassette can be introduced into a viral vector and transfected into the host cells transiently or permanently. For instance, the lentiviral vector can be engineered with interferon-α and Tie2 enhancer/promoter, co-expressing with miR-126/miR-130a to generate a hematopoietic stem/progenitor cells (HSC) stable cell line for cell-based therapy, in suppressing tumor growth and lung metastasis in vivo [466]. On the other hand, Trepel et al. modified miR-1d expressing AAV by changing tumor-targeted AAV capsid variant (ESGLSQS) and a non-specific cytomegaly virus (CMV) promoter to reduce cardiotoxicity and enhance the specificity to cancer [467]. The intravenous administering of modified vectors harboring the herpes simplex virus thymidine kinase (HSVtk) gene to PyMT mice, which is characterized by metastatic cancer growth, resulting in the suppression of multifocal breast tumors. Another recent study by Shu et al. also developed a retroviral system using a pSEBR-CimiR-vector consisting of a human elongation factor 1a-HIV enhancer hybrid promoter (hEFH) and the substitution of CimR with ts-miR to form a circular anti-miR-21 sponge [468]. This circularized anti-miR-21 sponge effectivity inhibits the oncogenic function of miR-231 and suppresses the tumor growth of the breast using the xenograft tumor model.

Non-viral systems can be further classified into three main sub-groups: polymeric, lipid- and inorganic-based carriers. The typical size of nanoparticles ranges from 1–150 nm and therefore, it can facilitate the efficient uptake of desired ligands by the cancer cells [474]. It also possesses tunable properties that allow the nanoparticles to be synthesized to variable coordination geometries that are of desire. In addition, the unique physiochemical properties of the nanoparticles exhibit a high stabilization in a reduced environment, as well as biocompatibility and resistance to endonuclease activity. Recent studies have demonstrated extensive efforts in modifying drugs to improve pharmacokinetics and enhancing delivery efficiency to target tissues. Among these, gold nanoparticle (AuNP) is of interest. AuNP is non-toxic in nature and with further modifications, it enables a higher dosage tolerance to target tissues [474]. It also possesses anti-cancer effects [475]. A study by Ramchandani et al., 2020 developed a tumor suppressive miR-708 mimetic conjugated with AuNP, which was layered with positively charged poly-L-lysine (PLL) followed by polyelectrolytes [469]. Using a mice model, miR-708-AuNP delivery led to a markedly fewer number of lung metastases. Histopathological analyses showed no toxicity to the heart, liver, kidneys, spleen, or bone marrow. Similarly, using serum biochemistry and immunogenic profiling, the study indicated the absence of renal, hepatic or muscle toxicity with no immunogenic effect. Another system is the use of the nano-lipid delivery system. The three disadvantages are the prolonged stability of the active pharmaceutical ingredients, low drug loading and drug release from liposomal delivery. However, the recent development of the lipid-based delivery system has overcome these limitations. For instance, Lujan et al., 2019 formulated a stable nanometer-sized liposome for miRNA encapsulation and facilitated its efficient transfer to breast cancer [470]. The nano-liposome was able to retain the small particle size under the condition of PBS, 10% FBS and ultrapure water. The delivery of the liposome enhanced the expression of miR-203 up to 40-fold. Another recent study by Yan et al. formulated a miRNA liposome by combining the functional liposomes (constituting of egg phosphatidylcholine (EPC), cholesterol, stearamide, and DSPE-PEG2000-tLyp-1) and miRNA complex consisting of tumor suppressor miRNA-203 and calf thymus DNA together [471]. Not only were the miRNA liposomes effectively internalized by MDA-MB-231 cells compared to the control, they also inhibited the invasion and migration by silencing the slug-mediated TGF-β/Smad pathway. The cytotoxicity profile also showed that the miRNA liposomal treatment did not affect the weight and no pathological abnormalities were observed in the major organs of the cancer-bearing mice. Another multifunctional nanocarrier is the polymer-based nanocarrier. A creatine-based nano-polymer POEG-PCre was synthesized by the reversible addition-fragmentation chain transfer (RAFT) co-polymerization of an OEG500 monomer and a vinylbenzyl chloride (VBC) monomer and creatine [472]. The co-delivery of DOX and tRNA-mir-34a using POEG-PCre nanocarrier was effectively transferred into the tumor. Treatment with DOX with tRNA-miR-34a synergized the anticancer effect by inducing apoptosis, necrosis and enhanced immune cell infiltration to the tumor bearing site in vitro and in vivo. Like the cytotoxic profile from previous studies, the co-delivery of DOX and tRNA-miR-34a did not induce much cytotoxic effect. These carriers have shown to be well tolerated by healthy normal cells while inducing anti-cancer effects.

## 6. Conclusions

The increasing evidences constantly reveal novel roles of miRNAs in regulating the hallmarks of breast cancers. Here, we described some of the potential miRNAs for miRNA-based therapeutics. Dysregulated miRNA signatures in different subtypes and cancer stages imply the importance of personalized therapy for the management of breast cancer. Recent advancement in developing delivery systems have been demonstrated to be non-invasive, effective, and as safe methods for miRNA-based therapies. Further studies are required to explore these miRNA potential candidates for therapeutic purposes and to unveil the critical underlying mechanisms regulated by these miRNAs to ensure its functional role in suppressing or enhancing tumor progression in breast cancer. With this, miRNA-based therapeutic strategies could be combined with conventional therapies, such as chemotherapy, endocrine therapy or targeted therapy, with the aim of enhancing or synergizing anti-cancer effects with reduced toxicity, and the improved overall survival rate of breast cancer patients.

## Figures and Tables

**Table 1:** Binding partners of miRNAs in regulating oncogenesis.

Function	miRNA	Target	Effects	Ref.
**Tumor suppressive (inhibition)**	miR-26a	RNF6	Proliferation	[53]
		CHD1, GREB1 and KPNA2		[54]
		Metadherin	Proliferation and metastasis	[55]
		MCL-1		[56]
		CCNE2	Sensitize to trastuzumab in Her2+ subtype	[57]
		ERBB2	Sensitize to tamoxifen in ER+ subtype	[58]
	miR-193a/b	DDAH1	Angiogenesis	[172]
		PTP1B	Proliferation and survival	[173]
		MORC4		[174]
		WT1	Proliferation and metastasis	[175]
		RAB22A		[176]
		MCL-1	Sensitize to doxorubicin	[177]
		uPA	Proliferation, cell invasion and metastasis	[178]
	miR-216a/b	TLR4	Suppresses stemness and the release of soluble factors associated with cancer-associated fibroblast activation	[194]
		PKCα	Survival and migration	[195]
		PAK2	Proliferation and metastasis	[196]
		SDCBP		[197]
		P2X7R	Proliferation and survival	[198]
		HDAC8	Proliferation and colony formation	[199]
	miR-223	Caprin-1	Proliferation and invasion	[203]
		STIM1		[204]
		HAX-1	Survival and sensitize to doxorubicin and cisplatin	[205]
		STAT5A	Survival and sensitize to paclitaxel	[206]
		EGF	Proliferation and sensitize radiation-treated breast cancer cells to lapatinib	[207]
**Oncogenic (promotion)**	miR-1246	CCNG2	Proliferation, migration, chemoresistance to docetaxel, epirubicin and gemcitabine	[456]

Abbreviations: Her2: human epidermal growth factor receptor 2; ER: estrogen receptor.

**Table 2:** Different types of miRNA-based therapies.

Treatment	Modes	Methods	miRNA	Types of Studies In Vivo/In Vitro	Ref
**miRNA inhibition therapy**	AMOs	2-OMe	miR-451	In vitro (MCF-7, SKBR3) In vivo (BALB/c)	[443]
			miR-21	In vitro (MCF-7 and HeLa)	[465]
	Gene editing	Crispr-cas9	miR-23b, miR-27b	In vitro (MCF7) In vivo (nude)	[314]
**miRNA replacement therapy**	Virus	Lentivirus	miR-126, miR-130a	In vitro (MDA-MB-231) In vivo (PyMT, C57Bl/6)	[466]
		Adeno-associated virus	miR-1d	In vitro (HEK293T and HeLa) in vivo (PyMT)	[467]
		Retroviral	miR-21	In vitro (SKBR3, MCF7, Jurkat, HEK-293T) In vivo (nude)	[468]
	Nanoparticle	Gold (PLL)	mir-708	In vitro (MDA-MB-231, HEK-293, 4T1) In vivo (CB-17 SCID and CB17.Cg-PrkdcscidHrhr/IcrCrl)	[469]
		Lipid	miR-203	In vitro (MDA-MB-231 and Hs578t cells)	[470]
			miR-203	In vitro (MDA-MB 231) In vivo (BALB/c)	[471]
	Polymer	Creatine	mir-34a	In vitro (4T1.2 and MDA-MB-231) In vivo (BALB/c)	[472]

Abbreviations: 2-OME: 2’-*O*-methylation; AMOs: anti-miR oligonucleotides; PPL: positively charged poly-L-lysine.
